# Combined BET and MEK Inhibition synergistically suppresses melanoma by targeting YAP1

**DOI:** 10.7150/thno.85437

**Published:** 2024-01-01

**Authors:** Rui Hu, Huihui Hou, Yao Li, Minghui Zhang, Xin Li, Yanzhong Chen, Ying Guo, Hongyin Sun, Shuang Zhao, Mengting Liao, Dongsheng Cao, Qin Yan, Xiang Chen, Mingzhu Yin

**Affiliations:** 1 Department of Dermatology, Hunan Engineering Research Center of Skin Health and Disease, Hunan Key Laboratory of Skin Cancer and Psoriasis, Xiangya Hospital, Central South University, Changsha, Hunan 410008, China.; 2 Department of Dermatology, The First Affiliated Hospital of Chongqing Medical University, Chongqing, China.; 3 Department of Pathology, Yale School of Medicine, New Haven, CT 06520, USA.; 4 Department of Medical Oncology, Harbin Medical University Cancer Hospital, Harbin, China.; 5 The first clinical college of Chongqing Medical University, Chongqing, China.; 6 Health Management of Xiangya Hospital, Central South University, Changsha, Hunan 410008, China.; 7 Xiangya School of Pharmaceutical Sciences, Central South University, Changsha, Hunan 410013, China.; 8 Clinical Research Center (CRC), Clinical Pathology Center (CPC), Cancer Early Detection and Treatment Center (CEDTC), Chongqing University Three Gorges Hospital, Chongqing University, Wanzhou, Chongqing, China.; 9 Translational Medicine Research Center (TMRC), School of Medicine Chongqing University, Shapingba, Chongqing, China.

**Keywords:** Melanoma, MEK inhibitor, BET inhibitor, YAP1, BRD4

## Abstract

**Rationale:** The response rate to the MEK inhibitor trametinib in BRAF-mutated melanoma patients is less than 30%, and drug resistance develops rapidly, but the mechanism is still unclear. Yes1-associated transcriptional regulator (YAP1) is highly expressed in melanoma and may be related to MEK inhibitor resistance. The purpose of this study was to investigate the mechanism of YAP1 in MEK inhibitor resistance in melanoma and to screen YAP1 inhibitors to further determine whether YAP1 inhibition reverses MEK inhibitor resistance.

**Methods:** On the one hand, we analyzed paired melanoma and adjacent tissue samples using RNA-seq and found that the Hippo-YAP1 signaling pathway was the top upregulated pathway. On the other hand, we evaluated the transcriptomes of melanoma samples from patients before and after trametinib treatment and investigated the correlation between YAP1 expression and trametinib resistance. Then, we screened for inhibitors that repress YAP1 expression and investigated the mechanisms. Finally, we investigated the antitumor effect of YAP1 inhibition combined with MEK inhibition both *in vitro* and *in vivo*.

**Results:** We found that YAP1 expression levels upon trametinib treatment in melanoma patients were correlated with resistance to trametinib. YAP1 was translocated into the nucleus after trametinib treatment in melanoma cells, which could render resistance to MEK inhibition. Thus, we screened for inhibitors that repress YAP1 expression and identified multiple bromodomain and extra-terminal (BET) inhibitors, including NHWD-870, as hits. BET inhibition repressed YAP1 expression by decreasing BRD4 binding to the YAP1 promoter. Consistently, YAP1 overexpression was sufficient to reverse the proliferation defect caused by BRD4 depletion. In addition, the BET inhibitor NHWD-870 acted synergistically with trametinib to suppress melanoma growth *in vitro* and *in vivo*.

**Conclusions:** We identified a new vulnerability for MEK inhibitor-resistant melanomas, which activated Hippo pathway due to elevated YAP1 activity. Inhibition of BRD4 using BET inhibitors suppressed YAP1 expression and led to blunted melanoma growth when combined with treatment with the MEK inhibitor trametinib.

## Introduction

Melanoma, the deadliest form of skin cancer, has a rising incidence and a five-year survival of only 27% for melanoma patients with distant metastasis 1. Immune checkpoint blockade, including anti-PD-1 and anti-CTLA-4 therapy, has brought effective and sustainable benefits to late-stage melanoma patients, with a response rate of approximately 30% 2, and MAPK inhibition therapy was reported to be effective in approximately 50% of melanomas with a BRAF mutation 3. Trametinib is an FDA-approved MEK inhibitor that benefits only a subset of melanoma patients 4, but the mechanisms of resistance are still unclear. Therefore, new therapeutics for use alone or in combination with MEK inhibitors are urgently needed to inhibit melanoma progression.

YAP1 (Yes1-associated transcriptional regulator) is a transcriptional coactivator that fuels several hallmarks of cancer 5, including tumor initiation, cell plasticity, drug resistance and metastasis 6. In melanoma patient samples, YAP1 is reported to be highly expressed due to copy number alteration, and its expression is correlated with poor prognosis 7. Several studies have revealed that YAP1 plays a critical role in melanoma proliferation and metastasis 8, 9, suggesting that YAP1 is a potential therapeutic target. Furthermore, high YAP1 levels have been shown to be correlated with resistance to trametinib in neuroblastomas 10. YAP1 depletion sensitizes neuroblastoma to trametinib, and overexpression of YAP1 induces trametinib resistance in neuroblastoma cells 10.

Emerging evidence has shown that epigenetic aberrations contribute to drug resistance. Among the epigenetic readers, the bromodomain and extra-terminal (BET) family proteins, which includes Bromodomain Containing 2 (BRD2), Bromodomain Containing 3 (BRD3), Bromodomain Containing 4 (BRD4) and Bromodomain testis-specific protein (BRDT), have been shown to be promising therapeutic targets in multiple types of cancer 11, 12. BRD2, BRD3 and BRD4 have been found to be expressed in a spectrum of tissues, while the expression of BRDT is limited to the testes 13. The two bromodomains recognize and bind to acetylated histone tails and promote transcriptional activation and elongation, which support uncontrolled proliferation by tumor cells 14. Recent studies have revealed that the functional target of BET proteins varies in different types of cancer 15-17; for example, p21 is the target in NSCLC 18, and AR is the target in prostate cancer 19. We previously reported the development of a novel and potent BET inhibitor, NHWD-870, aiming to improve the efficacy of BET inhibition in solid tumors, and NHWD-870 showed high potency and tolerable toxicity in vivo 20. NHWD-870 has entered a phase I clinical trial for multiple cancer indications in China (CXHL200250).

Here, we found that YAP1 expression was significantly increased after trametinib treatment in melanoma patients who did not respond to trametinib and correlated with poor survival of melanoma patients treated with trametinib. We also found that a MEK inhibitor mediated the translocation of YAP1 in melanoma cells, suggesting that YAP1 inhibition may sensitize melanoma to trametinib. Although there is no inhibitor that acts directly on YAP1 in use in the clinic because of the unstructured nature of YAP1 21, the combinatorial use of indirect inhibitors of YAP1 and MEK inhibitor may serve as an alternative avenue for melanoma treatment. In an effort to find YAP1 inhibitors, we screened 102 small molecules that were FDA-approved, in clinical trials, or druglike, and found that the BET inhibitor NHWD-870 strongly suppressed YAP1 expression and melanoma proliferation. Furthermore, the combination of a BET inhibitor and a MEK inhibitor synergistically suppressed melanoma progression, providing a promising therapeutic option for melanoma patients.

## Results

### Nuclear YAP1 expression is positively correlated with resistance to the MEK inhibitor trametinib and poor prognosis of melanoma patients

**Figure 1:**
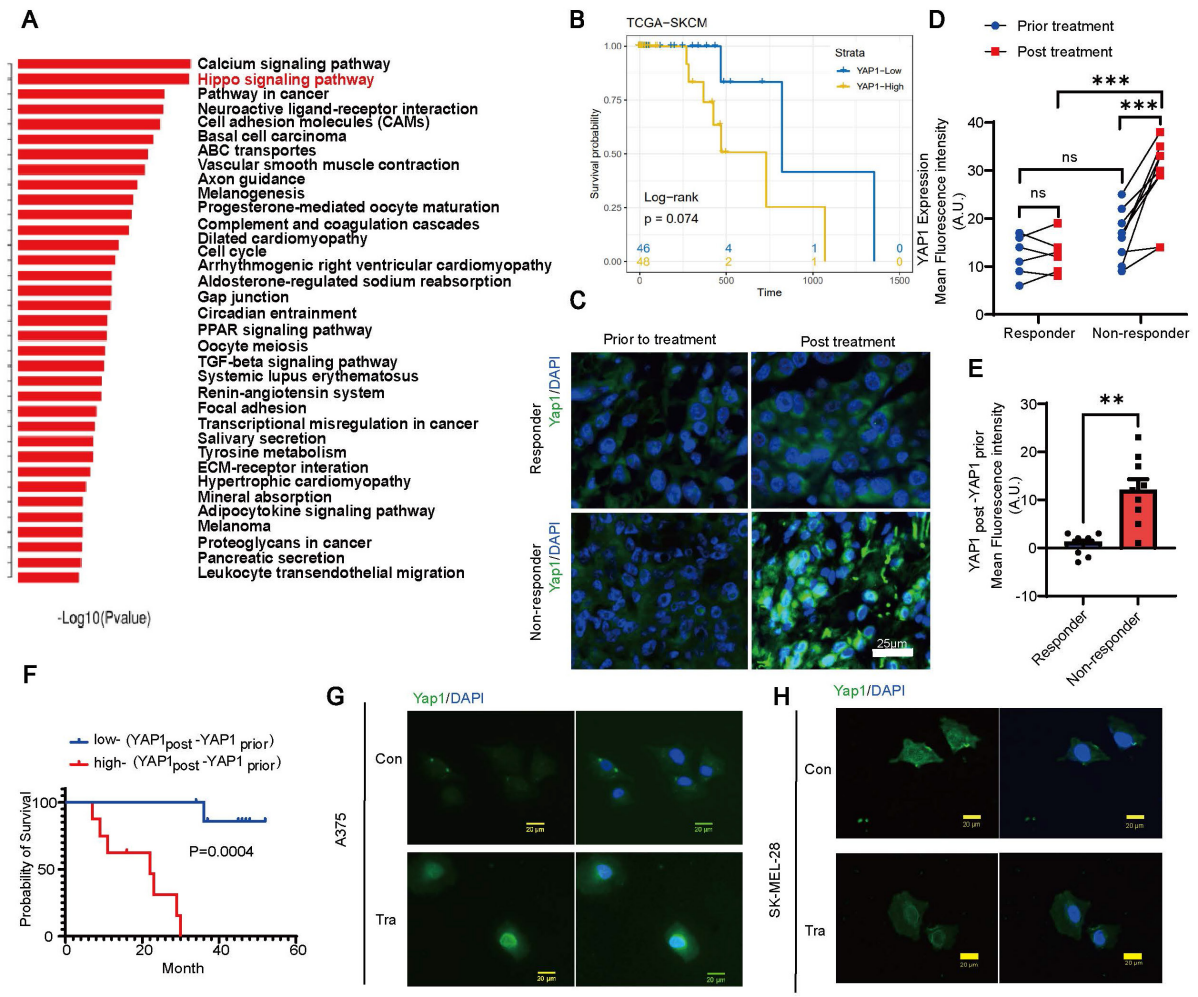
YAP1 expression is correlated with poor response to trametinib and survival of patients with melanoma. (A) Paired primary tumors and adjacent tissues from 23 melanoma patients were compared by using RNA-sequencing (RNA-seq) analysis. Shown are the pathways that were upregulated in the tumors. (B) Kaplan-Meier survival curve of two sub-groups that divided by YAP1 expression in TCGA-SKCM dataset. (C) YAP1 immunofluorescence (IF) staining of tumor tissues from responders and nonresponders before and after trametinib treatment. YAP1 (green) and DAPI (blue). (D) Fluorescence intensity of nuclear YAP1 expression in tumor tissue from responders and nonresponders before and after trametinib treatment. (E) Changes in YAP1 expression in tumor tissue from responders and nonresponders before and after trametinib treatment (YAP1_post_-YAP1_prior_). (F) Kaplan‒Meier analysis of the overall survival of melanoma patients treated with trametinib. The YAP1_post_-YAP1_prior_ high group had a worse prognosis than the YAP1_post_-YAP1_prior_ low group. (G-H) Representative images of YAP1 immunofluorescence (IF) staining in A375 cells (G) or SK-MEL-28 cells (H) before and after trametinib treatment. YAP1, green; DAPI, blue. * p < 0.05, ** p < 0.01, *** p < 0.001. The error bars represent the SEMs.

We analyzed paired melanoma and adjacent tissue samples from 23 patients 18 acral melanomas, 2 sun-exposed melanomas and 3 unclear-subset melanomas using RNA-seq and found that the Hippo-YAP1 signaling pathway was a top upregulated pathway in melanoma compared with paired adjacent tissues (Figure 1A). Furthermore, TCGA database analysis results showed that high expression of YAP1 seems to have poorer prognosis of skin cutaneous melanoma (SKCM) although P values showed no difference between high and low YAP1 expression (Figure 1B). Thus, we compared YAP1 expression before and after trametinib treatment. While there was no significant difference in nuclear YAP1 expression in responders before versus after treatment, trametinib treatment significantly increased nuclear YAP1 expression in nonresponders (Figure 1C-D). In addition, there was no significant difference in nuclear YAP1 expression between responders and nonresponders before trametinib treatment (Figure 1C-D), but nuclear YAP1 expression was significantly increased in nonresponders after trametinib treatment (Figure 1C). Moreover, the change in nuclear YAP1 expression after trametinib treatment was significantly greater in nonresponders than in responders (Figure 1E). Then, we correlated nuclear YAP1 expression with clinical outcome and found that the change in nuclear YAP1 expression was higher in patients with poor clinical outcomes than in those with good clinical outcomes before trametinib treatment (Figure S1A). Moreover, the change in nuclear YAP1 expression after trametinib treatment was significantly greater in patients with poor clinical outcomes than in patients with good clinical outcomes (Figure S1B). Consistently, Kaplan‒Meier analysis showed that the change in nuclear YAP1 expression after trametinib treatment was significantly correlated with poor prognosis in these melanoma patients (Figure 1F). To further investigate the role of YAP1 in melanoma progression, we analyzed YAP1 expression in the tumor stroma of patients who responded and nonresponded to trametinib treatment. The results showed that there was no difference in the expression of YAP1 in the stroma between nonresponders and responders before and after treatment (Figure S1C), and it was not related to the prognosis of patients (Figure S1D-F). These results prompted us to investigate whether YAP1 nuclear localization in melanoma cells changes after trametinib treatment. Immunofluorescence analysis showed that YAP1 was translocated into the nucleus after trametinib treatment in A375 and SK-MEL-28 melanoma cells (Figure 1G-H). Our findings suggested that YAP1 upregulation could account for the low sensitivity of MEK inhibition, consistent with the findings of a previous study 22. Thus, suppression of YAP1 might improve the therapeutic effect of trametinib in melanoma patients.

### YAP1 is necessary for melanoma growth

**Figure 2:**
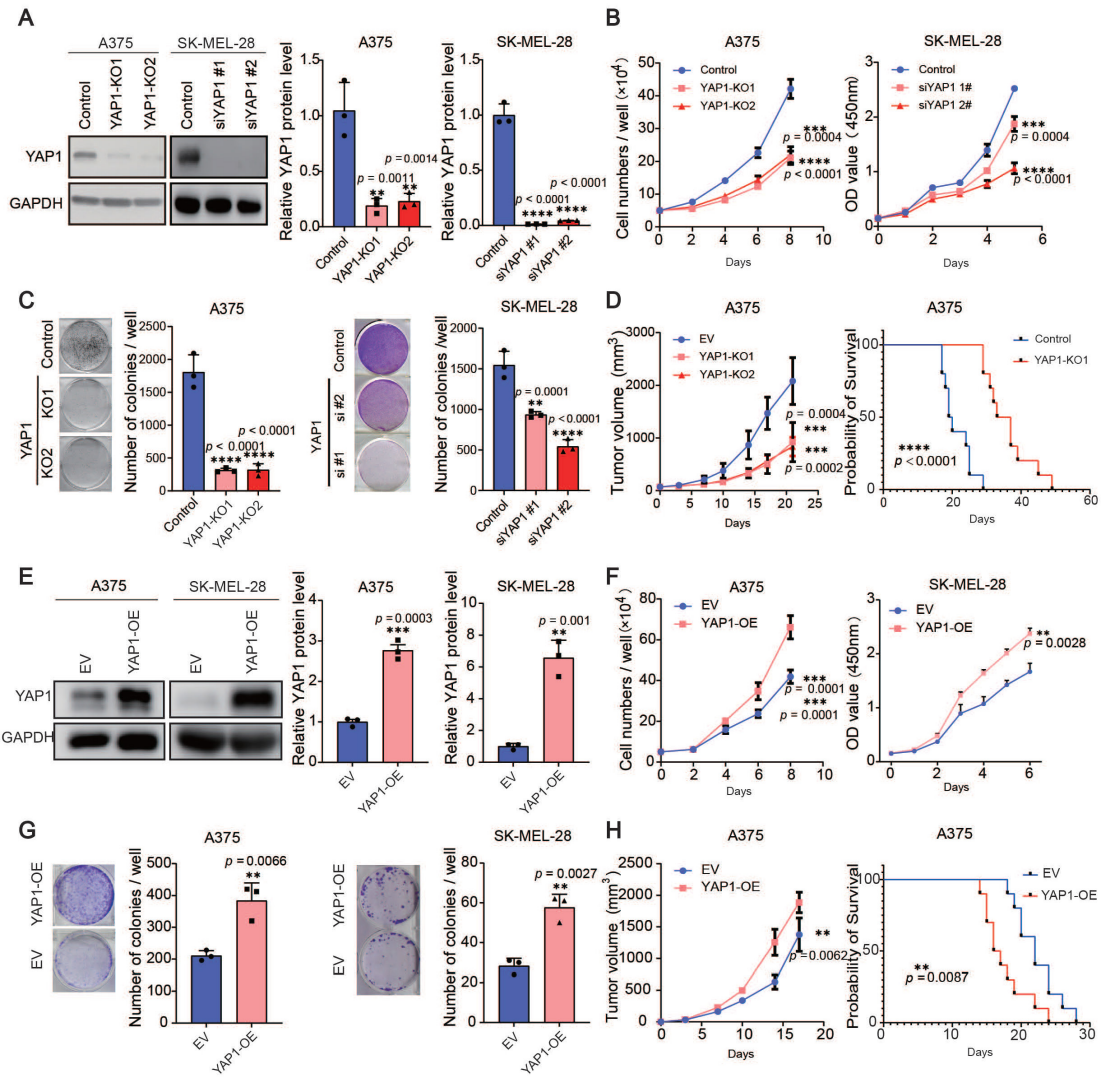
YAP1 plays an important role in melanoma growth. (A) Representative images (left) and quantification of western blot analysis (right) of control or YAP1-polyclonal knockout (KO1 or KO2) A375 and knockdown (siYAP1#1 or siYAP1#2) SK-MEL-28 cells. GAPDH served as the loading control. The data were selected from three independent experiments. (B-C) Cell counting assays (B) and colony formation assays (C) of control or YAP1-knockout (KO1 or KO2) A375 (left) and knockdown (siYAP1#1 or siYAP1#2) SK-MEL-28 (right) cells. (D) Tumor growth curves of nude mice implanted with control or YAP1-knockout (KO1 or KO2) A375 cells (n = 5/group) and Survival of mice after subcutaneous implantation of control or YAP1-knockout (KO1) A375 cells (n = 10/group). (E) Representative images (left) and quantification of western blot analysis (right) of control (EV) or YAP1 overexpression (OE) A375 and SK-MEL-28 cells. GAPDH served as the loading control. The data were selected from three independent experiments. (F-G) Cell counting assays (F) and colony formation assays (G) of control (EV) or YAP1 overexpression (OE) A375 and SK-MEL-28 cells. (H) Tumor growth curves of nude mice implanted with control (EV) or YAP1 overexpression (OE) A375 cells (n = 5/group) and Survival of mice after subcutaneous implantation of control or YAP1- overexpression (OE) A375 cells (n = 10/group). * p < 0.05, ** p < 0.01, *** p < 0.001. The error bars represent the SEMs.

The expression of YAP1 is correlated with stemness score, TOP2A and KI67 (Figure S2A), suggesting that YAP1 may be related to cell proliferation. Therefore, we used the CRISPR‒Cas9 system and siRNAs, respectively, to delete YAP1 in A375 cells and SK-MEL-28 cells (Figure 2A) and performed short-term proliferation assays as well as long-term colony formation assays (Figure 2B-C). Depletion of YAP1 in the A375 and SK-MEL-28 cell lines significantly inhibited the proliferation of melanoma cells. To further investigate the roles of YAP1, we injected A375 and YAP1 knockout cells into nude mice and measured their ability to form tumors. We found that YAP1 knockout blunted tumor growth and prolonged the survival of tumor-bearing mice (Figure 2D). Next, we established YAP1 overexpression in A375 and SK-MEL-28 (Figure 2E) cell lines for further validation. The results demonstrated that the overexpression of YAP1 in A375 and SK-MEL-28 significantly enhanced the proliferation of melanoma cells (Figure 2F-G). Moreover, the animal experiments corroborated these findings by revealing that YAP1 overexpression not only promoted tumor growth but also significantly reduced the survival rate of tumor-bearing mice (Figure 2H). These data suggest that YAP1 is necessary for melanoma growth *in vitro* and *in vivo*.

### Multiple BET inhibitors are inhibitors of YAP1 expression

**Figure 3:**
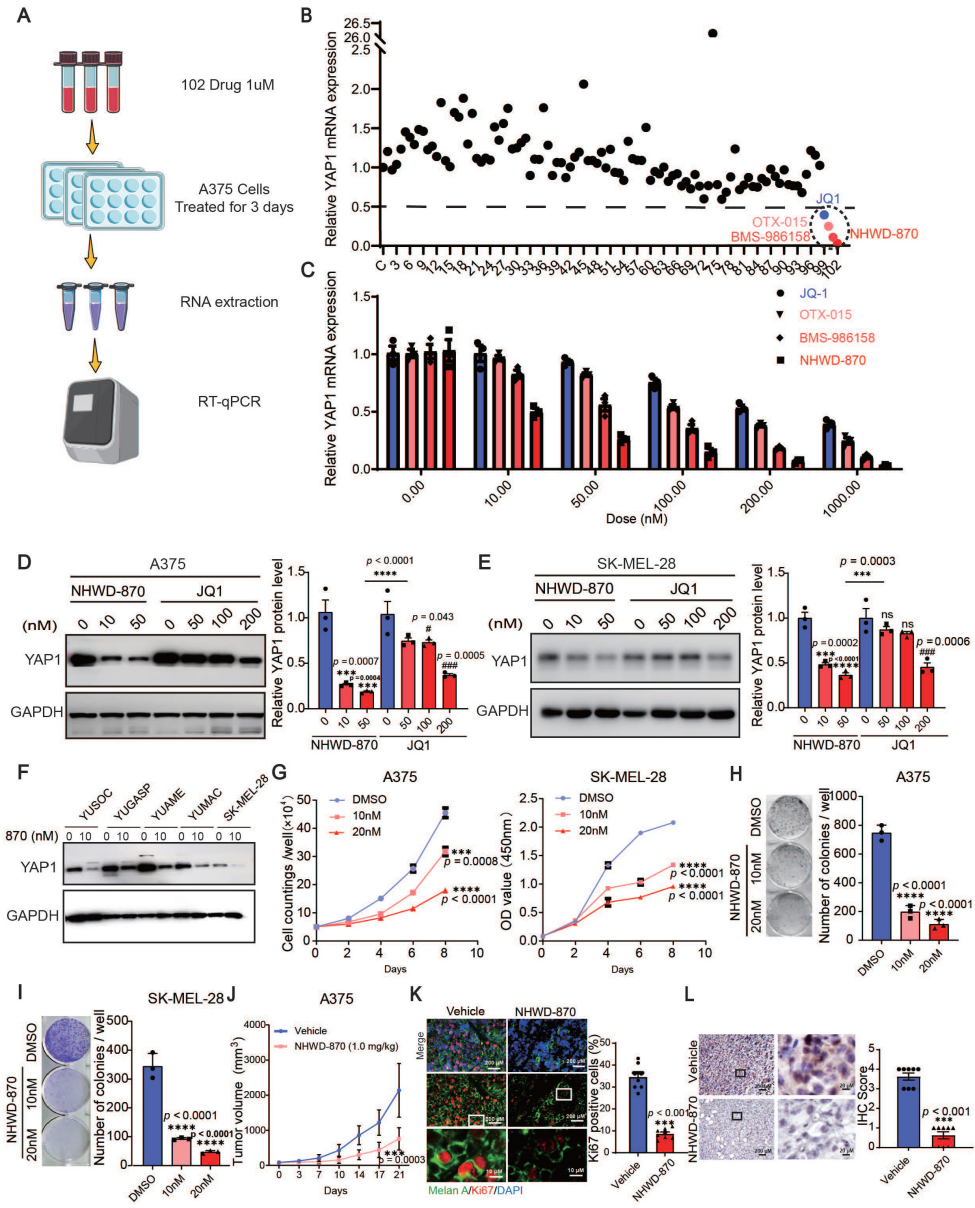
BET inhibitors inhibit YAP1 expression. (A) Flowchart of RT‒qPCR-based drug screening. (B) Results of drug screening for inhibitors of YAP1 expression in A375 cells. Screening with a concentration of 1 μM for 3 days identified 4 out of 102 small molecule compounds that decreased YAP1 expression by 50%. (C) Relative YAP1 mRNA levels in A375 cells treated with JQ1 and NHWD-870 at the indicated concentrations for 3 days. (D-E) Representative images (left) and quantification (right) of western blot analysis of A375 (D) and SK-MEL-28 (E) cells treated with JQ1 or NHWD-870 at the indicated concentrations for 3 days. (F) Relative YAP1 protein expression levels in YUSOC, YUGASP, YUAME, YUMAC and SK-MEL-28 melanoma cells treated with DMSO or 10 nM NHWD-870 for 3 days. (G) Cell counting assays of A375 (left) and SK-MEL-28 (right) cells cultured in DMSO or in 10 nM or 20 nM NHWD-870. (H-I) Representative images (left) and quantification (right) of colony formation assays of A375 (H) and SK-MEL-28 (I) cells treated with DMSO or with 10 nM or 20 nM NHWD-870. (J) Tumor growth curves for A375 tumor-bearing mice (n = 8/group) treated with vehicle or with 1 mg/kg NHWD-870 for 21 days. (K) Representative immunofluorescence images (left) and quantification (right) of Ki67+ cells in A375 tumors from mice treated with vehicle or with 1 mg/kg NHWD-870. Ki67, red; Melan A, green; and DAPI, blue. The scale bars are 200 μm and 10 μm. (L) Representative YAP1 staining (left) and IHC scoring (right) of A375 tumors from mice treated with vehicle or 1 mg/kg NHWD-870 (n = 8/group). The scale bars are 200 μm and 20 μm. * p < 0.05, ** p < 0.01, *** p < 0.001. The error bars represent the SEMs.

To target YAP1, we screened 102 small molecules that are FDA approved, currently in clinical trials, or druglike inhibitors of YAP1 expression in A375 cells. We found that multiple BET inhibitors, including NHWD-870, BMS-986158, OTX-015 and JQ1, were top hits that decreased YAP1 expression (Figure 3A-B). To further investigate the roles of BET inhibitors in melanoma, we performed RT‒qPCR and western blot analyses for YAP1 expression in A375 and SK-MEL-28 cells treated with different doses of NHWD-870 and JQ1. Both inhibitors decreased the mRNA and protein levels of YAP1 in a dose-dependent manner, and NHWD-870 showed a significantly stronger inhibitory effect than JQ1 at the same dose (Figure 3C-E). We also treated a panel of melanoma cells, including YUSOC, YUGASP, YUAME, YUMAC and SK-MEL-28 cells, with 10 nM NHWD-870 and found that YAP1 protein levels decreased in all these different melanoma cells (Figure 3F). NHWD-870 led to decreased proliferation and colony-forming ability of A375 and SK-MEL-28 cells in a dose-dependent manner *in vitro* (Figure 3G-I). We then treated A375 tumor-bearing mice with NHWD-870 and found that tumor growth was significantly attenuated *in vivo* (Figure 3J). Histological analysis revealed that Ki67 levels were decreased in tumors treated with NHWD-870 (Figure 3K). In addition, IHC analysis showed that YAP1 expression was decreased in tumors treated with NHWD-870 (Figure 3L). Together, these data suggest that BET inhibitors suppress melanoma proliferation by downregulating YAP1 expression.

### YAP1 is a direct downstream effector of BRD4 mediating melanoma proliferation

**Figure 4:**
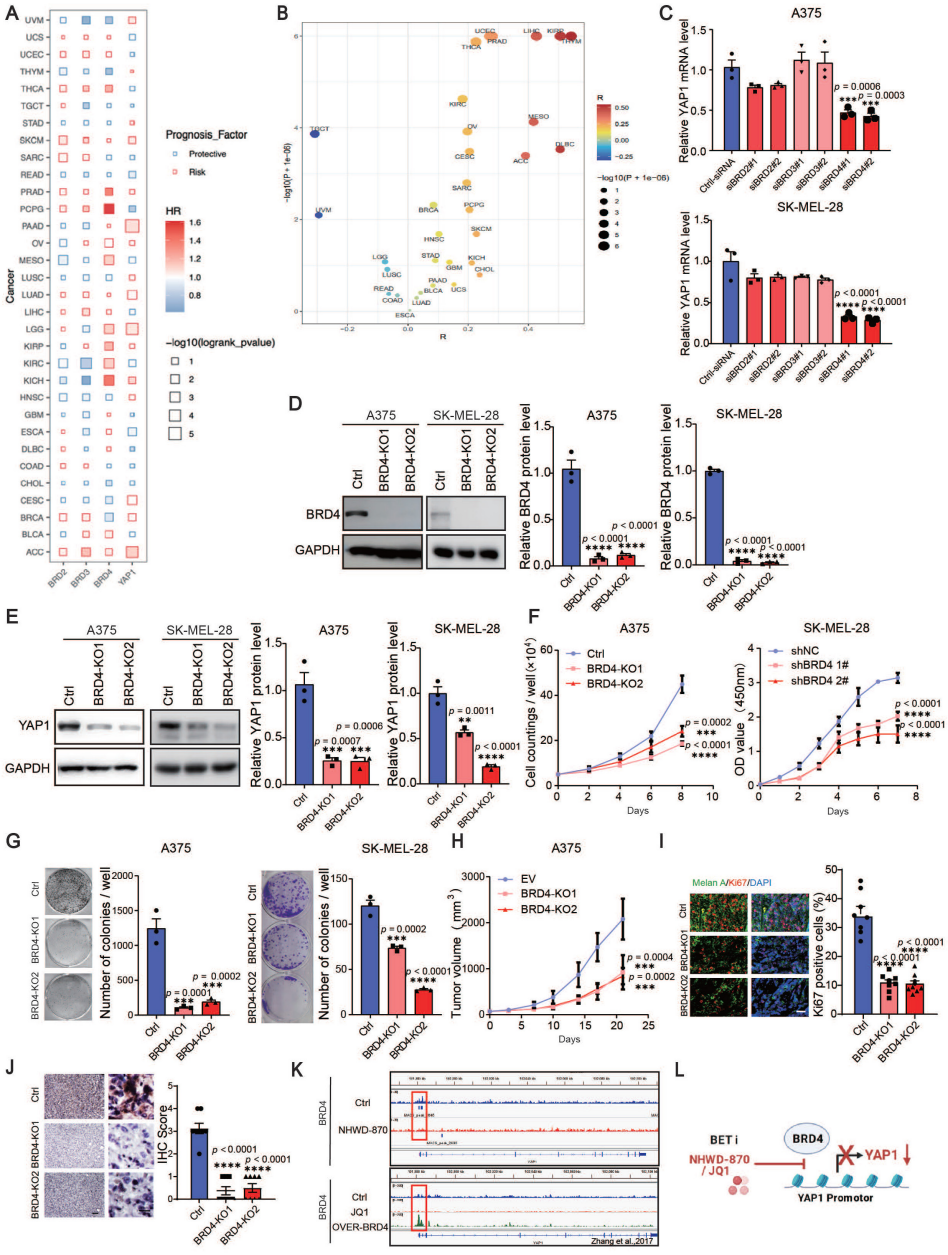
BRD4 promotes melanoma progression through direct regulation of YAP1. (A) The results of the association between overall survival time of patients and the expression of BRD2/BRD3/BRD4/YAP1 derived from Cox proportional-hazards model across pan-cancer datasets. (B) Spearman correlation coefficient (R, X axis) and P value (Y axis) of the association between YAP1 and BRD4 across pan-cancer datasets, each point represents a cancer type. (C) Relative YAP1 mRNA levels in A375 (top) and SK-MEL-28 (bottom) cells transfected with siRNA (ctrl-siRNA, siBRD2#1, siBRD2#2, siBRD3#1, siBRD3#2, siBRD4#1, siBRD4#2). (D) Representative western blots (left) and quantification (right) western blot analysis of BRD4 protein levels of control (Ctrl) or BRD4-knockout (KO1 or KO2) A375 and SK-MEL-28 cells. (E) Representative western blots (left) and quantification (right) western blot analysis of YAP1 protein levels of control or BRD4-knockout (KO1 or KO2) A375 and SK-MEL-28 cells. (F) Cell counting assays of control or BRD4-knockout (KO1 or KO2) A375 (left) and SK-MEL-28 (right) cells. (G) Representative images (left) and quantification (right) of colony formation assays of control (Ctrl) or BRD4-knockout (KO1 or KO2) A375 (left) and SK-MEL-28 (right) cells. (H) Tumor growth curves of control (Ctrl) or BRD4-knockout (KO1 or KO2) A375 tumor-bearing mice 21 days after subcutaneous implantation in nude mice (n = 8/group). (I) Representative immunofluorescence images (left) and quantification (right) of Ki67+ melanoma cells in control (Ctrl) or BRD4-knockout (KO1 or KO2) A375 tumors. Ki67, red; Melan A, green; and DAPI, blue. The scale bar is 50 μm. (J) Representative YAP1 staining (left) and IHC scoring (right) of control (EV) or BRD4-knockout (KO1 or KO2) A375 tumors. The scale bar is 50 μm. (K) Genome browser views of BRD4 ChIP-seq peaks on the *YAP1* promoter: BRD4 ChIP of A375 cells treated with DMSO or 4 nM NHWD-870 for 3 days (top panel) and DMSO-treated, JQ-1-treated, or BRD4-overexpressing cells (bottom panel). (L) Model showing the effects of the BET inhibitors JQ1 and NHWD-870 on BRD4 regulation of YAP1. * p < 0.05, ** p < 0.01, *** p < 0.001. The error bars represent the SEMs.

The elevated expression of BRD2/BRD3/BRD4/YAP1 is a risk factor from the Cox cross-pan-cancer dataset proportional hazards model in a variety of tumors, including SKCM (Figure 4A), besides, there was an association between BRD4 and YAP1 (Figure 4B). To further verify the possible mechanisms through which BET inhibitors suppress YAP1 expression, we knocked down BRD2, BRD3 and BRD4 in A375 and SK-MEL-28 cells with siRNAs. We found that only BRD4 knockdown decreased the expression of YAP1 (Figure S3A-B and Figure 4C). Using the CRISPR‒Cas9 system, we found that deletion of BRD4 in A375 and SK-MEL-28 cells recapitulated the effects of BET inhibitors (Figure 4D-E). Cell proliferation assays and clonogenic assays also showed that BRD4 knockout decreased melanoma proliferation (Figure 4F-G). Consistently, BRD4 deletion slowed the growth of A375 tumors in nude mice (Figure 4H). Histological analysis revealed that Ki67 and YAP1 expression was decreased in BRD4-knockout tumors (Figure 4I-J). These data suggest that BRD4 strongly decreases YAP1 expression and melanoma growth. To determine whether BRD4 directly regulates YAP1, we performed ChIP-seq analysis and found a BRD4-binding peak on the *YAP1* promoter in A375 cells. This peak was decreased in A375 cells treated with NHWD-870 (Figure 4K top panel). These data were consistent with the Zhang laboratory's ChIP-seq data, which showed that BRD4 binding to the YAP1 promoter was attenuated by JQ1 treatment and enhanced in BRD4-overexpressing cells (Figure 4K bottom panel) 23. These results indicate that BRD4 promotes melanoma progression through direct regulation of YAP1 (Figure 4L).

**Figure 5:**
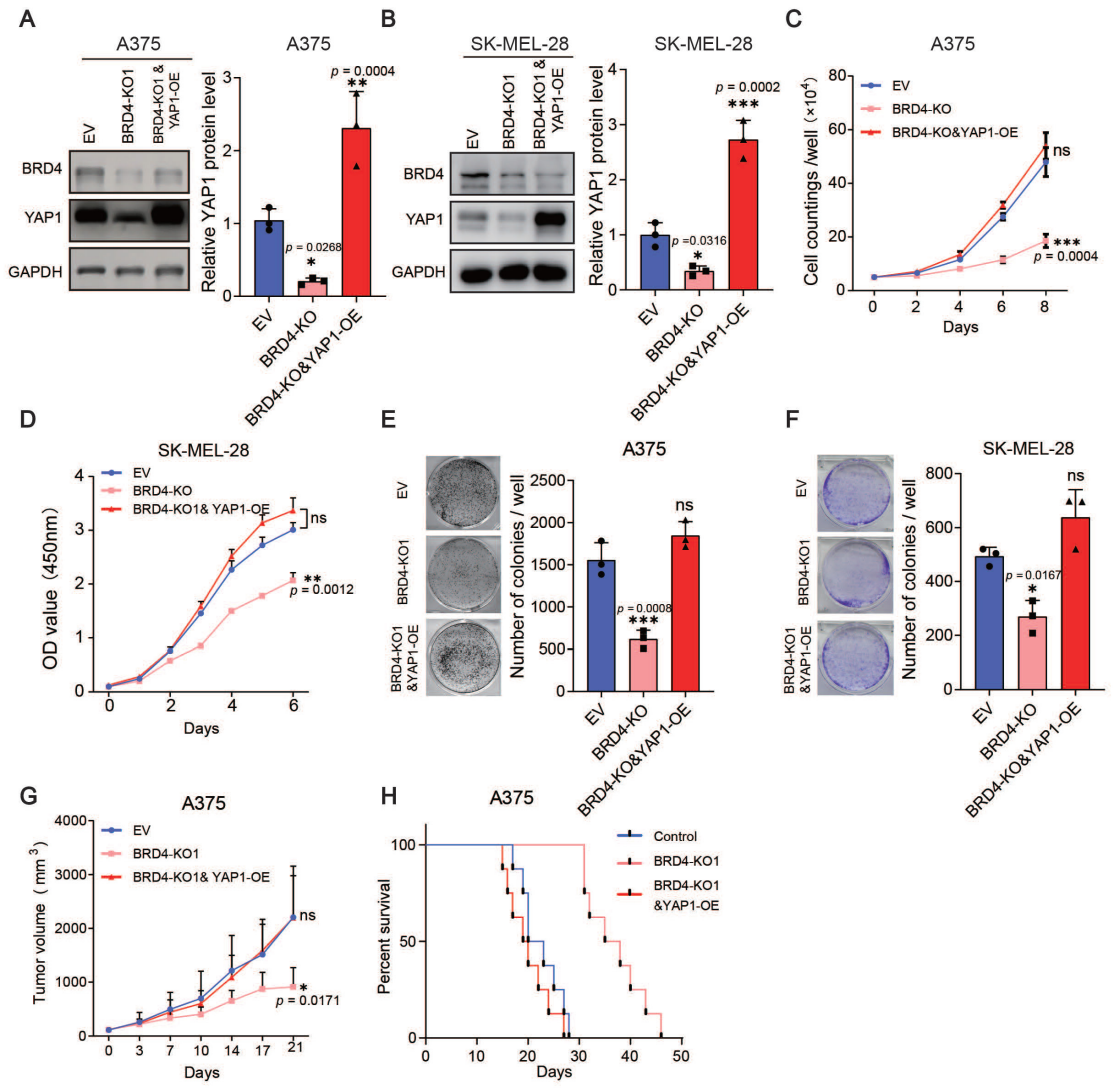
Overexpression of YAP1 rescues the proliferation defects caused by BRD4 knockout. (A-B) Representative western blots (left) and quantification (right) of western blot analysis of BRD4 and YAP1 protein levels in control A375 (A) and SK-MEL-28 (B) cells, A375 (SK-MEL-28) cells with BRD4 KO, or A375 (SK-MEL-28) cells with BRD4 KO and YAP1 overexpression. (C-D) Cell counting assays of control A375 (C) and SK-MEL-28 (D) cells, A375 (C) and SK-MEL-28 (D) cells with BRD4 KO, or A375 (C) and SK-MEL-28 (D) cells with (BRD4 KO and YAP1 overexpression. (E-F) Representative images (left) and quantification (right) of colony formation assays of control A375 (E) and SK-MEL-28 (F) cells, A375 (E) and SK-MEL-28 (F) cells with BRD4 KO, or A375 (E) and SK-MEL-28 (F) cells with BRD4 KO and YAP1 overexpression. (G) Tumor growth curves of nude mice subcutaneously implanted with control A375 cells, A375 cells with BRD4 KO, or A375 cells with BRD4 KO and YAP1 overexpression (n = 6/group). (H) Survival of nude mice after subcutaneous implantation of control A375 cells, A375 cells with BRD4 KO, or A375 cells with BRD4 KO and YAP1 overexpression (n = 8/group). * p < 0.05, ** p < 0.01, *** p < 0.001. The error bars represent the SE = M.

Furthermore, we overexpressed YAP1 in the BRD4-knockout A375 and SK-MEL-28 cell lines (Figure 5A-B) and performed proliferation assays and xenograft studies. The overexpression of YAP1 completely reversed the anti-proliferation phenotype of BRD4 knockout both *in vitro* and *in vivo* (Figure 5C-H). Together, these data indicate that the BRD4-YAP1 axis is critical for melanoma proliferation.

### Combined BET and MEK inhibition synergistically suppresses melanoma growth

**Figure 6:**
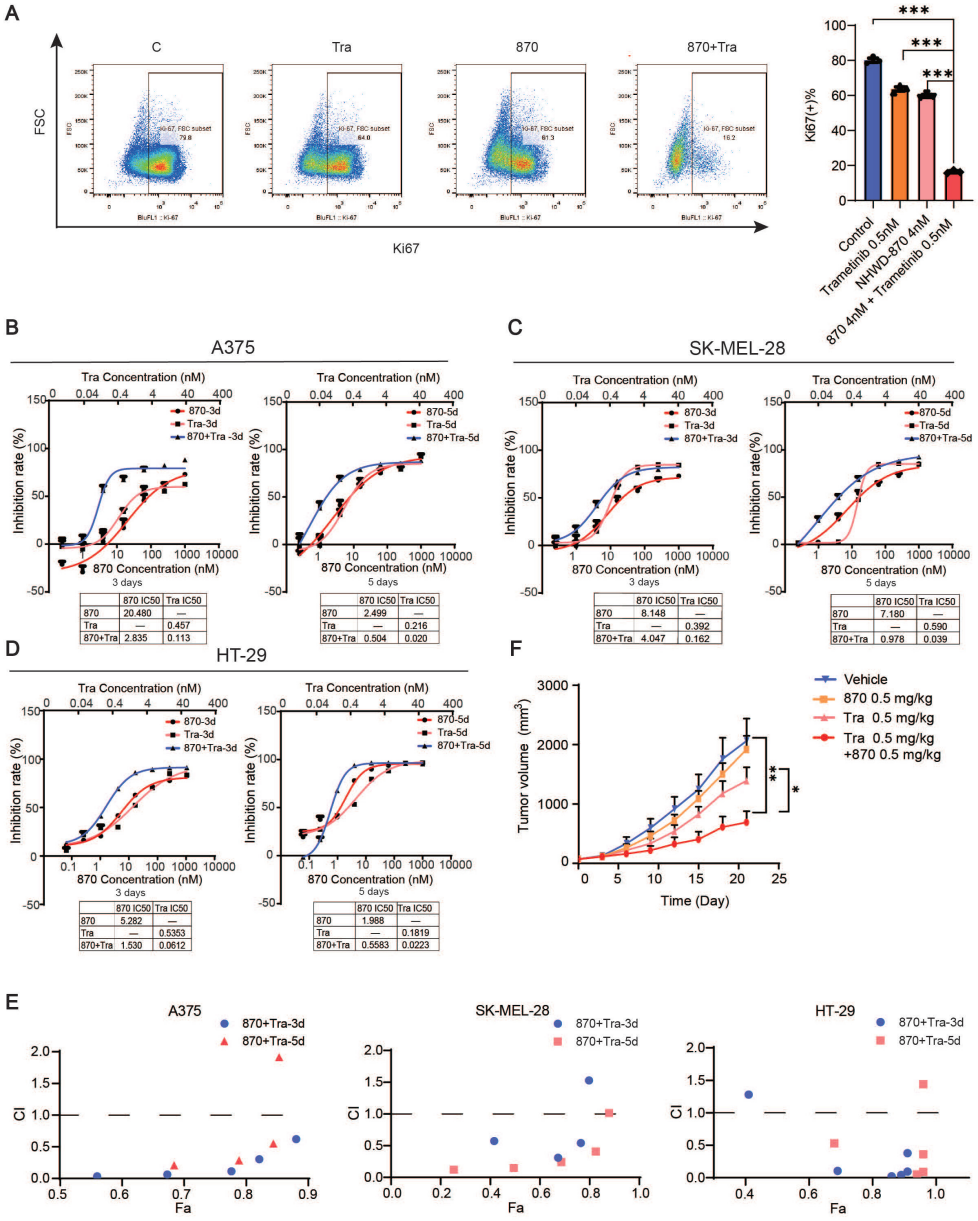
Combined BET and MEK inhibition synergistically suppresses melanoma growth. (A) Representative flow cytometry plots (left) and quantification (right) of Ki67+ cells in A375 cells treated with trametinib and/or NHWD-870 for 3 days. (B-D) Inhibition effect and IC50s of trametinib and NHWD-870 combination in A375 (B), SK-MEL-28 (C) and HT-29 (D) cells. Tra, trametinib; 870, NHWD-870. (E) Combination treatment of trametinib and NHWD-870 depicted as the Fa-CI (fraction affected—combination index) plot shows synergy between the two drugs in the A375 (left), SK-MEL-28 (center) and HT-29 (right) cells. CI values of 0.1-0.3, 0.3-0.7, 0.7-0.85, and 0.85-0.90 indicate strong, medium, modest and slight synergism, respectively. CI values of 0.90-1.10 indicate nearly additive effects, and those >1.1 indicate antagonism. 8T, NHWD-870 plus trametinib. (F) Tumor growth curves of nude mice subcutaneously implanted with A375 cells and treated with 0.5 mg/kg trametinib in combination with 0.5 mg/kg NHWD-870 (n = 6-7/group). T, trametinib; 870, NHWD-870. * p < 0.05, ** p < 0.01, *** p < 0.001. The error bars represent the SEMs.

We next investigated the efficacy of the combination of BET and MEK inhibition. We treated melanoma cell line A375, SK-MEL-28 and colorectal cancer cell line HT29 with NHWD-870 and trametinib *in vitro*, and flow cytometry analysis revealed that Ki67 expression was strongly decreased in the combination group (Figure 6A). Moreover, we found that the combination synergistically inhibited melanoma cell growth as measured by CCK-8 cell proliferation assays (Figure 6B-D). Combination index (CI) analysis and normalized isobolograms for trametinib and NHWD-870 combination further supported the synergistic nature of this combination (Figure 6E and Figure S4). To test its efficacy *in vivo*, we treated A375 tumor-bearing nude mice with NHWD-870, trametinib or their combination. Consistent with our *in vitro* findings, NHWD-870 and trametinib acted in synergy *in vivo* (Figure 6F).

### BRD4 expression is correlated with YAP1 expression, lack of response, and poor survival of melanoma patients treated with the MEK inhibitor trametinib

**Figure 7:**
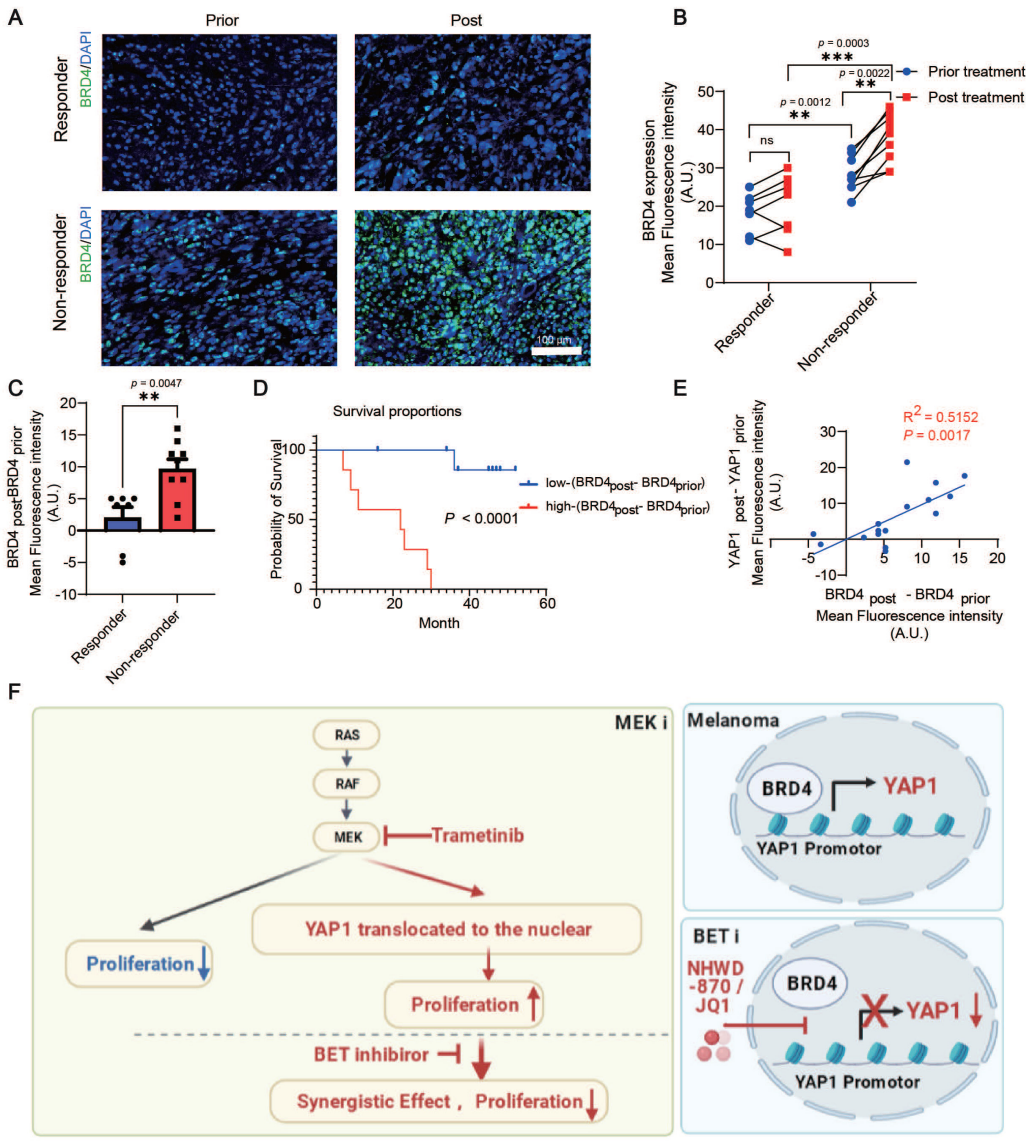
BRD4 expression is correlated with YAP1 expression and poor response and survival of patients with melanoma treated with the MEK inhibitor trametinib. (A) Representative immunofluorescence staining of tumor tissue from responders and nonresponders before and after trametinib treatment. BRD4, green; DAPI, blue. (B) BRD4 expression in tumor tissue from responders and nonresponders before and after trametinib treatment. (C) Change in BRD4 expression (BRD4_post_-BRD4_prior_) in tumor tissue from responders and nonresponders before and after trametinib treatment. (D) Kaplan‒Meier analysis of overall survival. The BRD4_post_-BRD4_prior_ high group had a worse prognosis in terms of survival than the BRD4_post_-BRD4_prior_ low group. (E) The change in BRD4 expression (BRD4_post_-BRD4_prior_) is positively correlated with the change in YAP1 expression (YAP1_post_-YAP1_prior_). (F) Working model summarizing the major findings. * p < 0.05, ** p < 0.01, *** p < 0.001. The error bars represent the SEMs.

We analyzed BRD4 expression in patients with melanoma before or after trametinib treatment. Consistent with the change in YAP1 expression, there was no significant difference in BRD4 expression in responders before versus after treatment. However, trametinib treatment significantly increased BRD4 expression in nonresponders but not in responders (Figure 7A-B). Our results also showed that BRD4 expression in nonresponders was higher than that in responders before treatment (Figure 7B). In addition, the increase in BRD4 expression (BRD4_post_-BRD4_prior_) was higher in nonresponders after trametinib treatment than in responders (Figure 7C). Consistently, BRD4 expression in patients with poor clinical outcomes was higher than that in patients with good clinical outcomes before trametinib treatment (Figure S5A). In addition, the change in BRD4 expression was significantly elevated after trametinib treatment in the poor-clinical-outcome group compared with the good-clinical-outcome group (Figure S5B). Furthermore, the change in BRD4 expression (BRD4_post_-BRD4_prior_) was positively correlated with the poor prognosis of patients with melanoma treated with trametinib (Figure 7D). The change in BRD4 expression was also positively correlated with the change in YAP1 expression (p = 0.0017, R^2^ = 0.5152) (Figure 7E). These results indicate that both YAP1 expression and BRD4 expression are correlated with the response and the outcome of trametinib treatment in patients with melanoma.

## Materials and methods

### Compounds

NHWD-870 and BMS-986158 were synthesized by Ningbo Wenda Pharma (Ninghai, Zhejiang, China) and donated. JQ1 was purchased from MedChem Express (Cat # HY-13030). The other 101 small molecules were purchased from Selleck (Table S1).

### Cell culture

A375 and SK-MEL-28 cells were obtained from the ATCC (Manassas, USA). Both were cultured in DMEM supplemented with 10% FBS, 100 U/mL penicillin and 100 mg/mL streptomycin. YUSOC, YUGASP, YUAME, and YUMAC are cell lines derived from tumors of patients treated at Yale University and were grown in Opti-MEM plus with 5% FBS.

### Clinical samples

All clinical specimens in this study were collected with informed consent for research use and were approved by Central South University Institutional Review Boards in accordance with the Declaration of Helsinki. Melanoma tumor specimens were excised to alleviate tumor burden. The data referenced in this study are available under GSE190113 in the Gene Expression Omnibus (GEO). After institutional review board approval, we collected melanoma tissues from patients who met our inclusion criteria from January 2017 to December 2020 in the Tumor Hospital of Harbin Medical University. All the patients enrolled for the study were ≥18 years and had histologically confirmed unresectable stage IIIC or IV melanoma with a mutation at the 600th position in BRAF. “Good outcome” and “Poor outcome” means the patients were still alive or dead when we collected the date in May, 2021.

### Immunofluorescence study

Immunofluorescence staining of melanoma tumor sections was performed according to the manufacturer's instructions. Quantitative comparison of YAP1 (ab56701, Abcam,1:400), and BRD4 (#13440, CST 1:200) expression was performed using freeware image analysis software (ImageJ, WS Rasband, National Health Institute, Bethesda, MA, USA) as previously reported 24. The cell area was determined by manual delineation of raw fluorescence images. A minimum of 12 cells were analyzed from two independent experiments.

### CRISPR/siRNAs knockout/knockdown and YAP1 overexpression

Knockout sgRNAs were designed according to online software CHOPCHOP (https://chopchop.cbu.uib.no/) and cloned into LentiCRISPRv2 vector. lentiviral plasmid, psPAX2, and pMD2.G were transfected into HEK293T cells in 6-well plates by Turbofect transfection Reagent (R0532, Thermo Fisher Scientific). The supernatants were collected and filtered (SLHV033RS, Millipore) 48 h after transfection. Lentiviruses were used for melanoma cell infection. Melanoma cells with stable knockdown or overexpression were selected with 1 μg/ml puromycin. YAP1-V5 in pLX304 (Addgene #25890) was used for YAP1 overexpression. Duplexes of siRNA were synthesized by Genepharma (Shanghai, China). Transfection of siRNA was performed according to manufacturer's instructions. Non-targeting siRNA was used as a control.

The sequences of the sgRNAs were as follows:

BRD4: 1: 5'-AGACCAACCAACTGCAATACCT-3' and 2: 5'-GAGTCTGGGATGTTCGTCTCTC-3'; and YAP1: 1: 5'-GTGCACGATCTGATGCCCGG-3' and 2: 5'-ACATCGATCAGACAACAACA-3'.

The sequences of the siRNAs were as follows:

YAP1: 1: sense 5'-CUGCCACCAAGCUAGAUAATT-3', anti-sense 5'-UUAUCUAGCUUGGUGGCAGTT-3'; and YAP1: 2: sense 5'-GCAUCUUCGACAGUCUUCUTT-3', anti-sense 5'-AGAAGACUGUCGAAGAUGCTT-3'.

### RT‒qPCR

Total RNA was isolated with TRIzol reagent (15596026, Invitrogen) according to the manufacturer's instructions. cDNA was synthesized with HiScript® II Q RT SuperMix for qPCR (+gDNA Wiper) following the manufacturer's instructions (R223-01, Vazyme). RT-qPCR was performed with 2X SYBR Green qPCR Master Mix (B21703, Bimake) using an ABI QuantStudio 3 PCR system. The expression of RNA is shown relative to the level of GAPDH mRNA. The primers used for RT‒qPCR were as follows:

BRD2 F' GAGGTGTCCAATCCCAAAAAGC

BRD2 R' ATGCGAACTGATGTTTCCACA

BRD3 F' TCAAATTGAACCTGCCGGATT

BRD3 R' TGCATACATTCGCTTGCACTC

BRD4 F' CGCTATGTCACCTCCTGTTTGC

BRD4 R' ACTCTGAGGACGAGAAGCCCTT

GAPDH F' CTCTGCTCCTCCTGTTCGAC

GAPDH R' GCCCAATACGACCAAATCC

YAP1 F' TAGCCCTGCGTAGCCAGTTA

YAP1 R' TCATGCTTAGTCCACTGTCTGT

### Western blot assay

Whole-cell lysates were extracted with RIPA lysis buffer according to the manufacturer's protocol. Proteins were separated on a 10% SDS‒PAGE gel and identified by the following antibodies: an α-Tubulin (11H10) rabbit mAb (#2125, CST), an anti-YAP1 antibody (ab56701, Abcam), a c-Myc antibody (9E10, Novus Biologicals), a GAPDH (D4C6R) mouse mAb (#97166, CST), and a BRD4 (E2A7X) rabbit mAb (#13440, CST).

### ChIP-seq

Chromatin immunoprecipitation (ChIP)-seq was performed by Acegen. Briefly, A375 cells were treated with 4 nM NHWD-870 for 3 days. Then, those cells were collected and cross-linked in DMEM with 1% formaldehyde. A Bioruptor was used to sonicate chromatin, after which incubation was performed with a BRD4 antibody (#13440, CST) for immunoprecipitation. The DNA library was prepared using an Acegen DNA Library Prep Kit from Illumina, amplified by twelve-cycle PCR, cleaned up, analyzed with an Agilent 2100 Bioanalyzer and finally sequenced on the Illumina platform.

### Colony formation assay and cell proliferation assay

For the colony formation assay, A375 cells or A375-derived cells were seeded into 6-well plates at 1000 cells per well. Seven days later, 10% formalin was used to fix the cells for 20 minutes at room temperature. Then, they were stained with 0.05% crystal violet in distilled water for 1 hour. Afterward, they were washed with water 3 times. Pictures were captured using a scanner. A CCK-8 assay was used to assess cell proliferation. A375 cells or SK-MEL-28 cells were seeded into 96-well plates at 800 cells per well and incubated for 1-5 days. At various time points, the medium was replaced with fresh medium containing 10% CCK-8 reagent (Bimake, Cat# B34302, USA). After incubation for 2.5 h, the absorbance at 450 nm was measured. For combinational treatments, the combination index (CI) was calculated using CompuSyn software (ComboSyn Inc., Biosoft; Cambridge, UK).

### Ki67-positive cell analysis

A375 cells were seeded into 6-well plates at 5 × 10^5^ cells per well. After 12 h, the cells were treated with NHWD-870 and/or trametinib. After incubation for 3 days, an anti-Ki67-FITC antibody (Thermo Fisher Scientific, #11-5698-80) was used to stain the cells. Ki67-positive cells were detected by flow cytometry and analyzed using FlowJo software.

### Animal studies

A total of 1x10^6^ A375- or A375-derived cells were subcutaneously implanted into the flanks of female nude mice. For drug treatment, when the tumors reached approximately 100 mm^3^, 0.5 mg/kg NHWD-870 once daily (5 days on, 2 days off) and/or 0.5 mg/kg trametinib once daily or vehicle was administered to the mice orally 20, 25, 26. The tumor sizes were measured every 3 or 4 days using a caliper. All mice were sacrificed by cervical dislocation after intraperitoneal injection with 0.1% pentobarbital sodium (30 mg/kg), and the tumors were dissected and fixed in formalin. All animals were housed in the specific pathogen-free animal facilities of Central South University with a 12:12 h light-dark cycle at a constant room temperature (22 ±1 °C) and fed a standard laboratory diet.

## Conclusion

In summary, we identified a new vulnerability for MEK inhibitor-resistant melanomas, which have an activated Hippo pathway due to elevated YAP1 activity. Inhibition of BRD4 using BET inhibitors suppressed YAP1 expression and led to blunted melanoma growth when combined with treatment with the MEK inhibitor trametinib. Thus, these results suggest a promising combination therapy strategy for melanoma treatment.

### Ethics approval and consent to participate

The study was approved by the Medical Ethics Committee of Xiangya Hospital of Central South University (permit number: 202004337). The animal study was approved by Animal Ethics Committee of Central South University Institutional Review Boards (permit number: 2020sydw0679).

### Bioinformatics analysis of pan-cancer datasets from TCGA project

#### Data collection

The gene expression profile and the corresponding clinical data of 33 cancer types from The Cancer Genome Atlas (TCGA) research network were collected using TCGAbiolinks R package. In our study, we only considered primary tumor samples and using TCGA barcode (https://docs.gdc.cancer.gov/Encyclopedia/pages/TCGA_Barcode/) to retain them.

#### Exploring the association between YAP1 and tumor proliferation in cutaneous melanoma

Signature of stemness was downloaded from MSigDB website (http://www.gsea-msigdb.org/gsea/msigdb/cards/MALTA_CURATED_STEMNESS_MARKERS). We calculated stemness score across cutaneous melanoma (TCGA-SKCM) samples using GSVA R package. After that, the association between YAP1 expression and stemness score were determined using Spearman correlation test. Besides, we also tested the association between YAP1 and two proliferation related genes (MKI67 and TOP2A) based on Spearman correlation test.

#### Assessment of the correlation between YAP1 and BRD4 protein across pan-cancer datasets

The correlation between YAP1 and BRD4 protein was obtained using Spearman correlation test across 33 cancer types. Specifically, the correlation coefficient (R) and P value were used for downstream visualization.

### Survival analysis

The association between the overall survival time of patients and the expression of investigated genes (YAP1, BRD2, BRD3, BRD4) were explored based on Cox proportional-hazards model. Genes with hazard ratio (HR) greater than 1 were regarded as risk factors, on the contrary, those with HR less than 1 were considered as protective factors. Kaplan-Meier survival curve was plotted between sub-groups that divided by the median of gene expression value. The Cox proportional-hazards model and Kaplan-Meier survival curve were implemented in survival R package.

### Statistical analysis

All data from the experiments are expressed as the mean ± SEM. The data were statistically analyzed with an unpaired Student's t test or one-way ANOVA/Tukey's multiple comparisons test using GraphPad Prism 5 (GraphPad Software, La Jolla, CA). Overall survival (OS) was analyzed using the Kaplan-Meier method. Statistical significance was set at *P < 0.05, **P < 0.01, and ***P < 0.001.

## Discussion

In this study, we first identified the Hippo-YAP signaling pathway as a top elevated pathway in patients with melanoma. Analysis of YAP1 expression in melanoma patients treated with trametinib showed that YAP1 expression was negatively correlated with the response and survival in these patients. Consistently, YAP1 was enriched in the nucleus after trametinib treatment in melanoma cells, suggesting that increased nuclear YAP1 could lead to resistance to MEK inhibition (Figure 7F). Therefore, we screened for inhibitors of YAP1 expression and identified multiple BET inhibitors as the top hits. Mechanistically, BRD4 binds the *YAP1* promoter to activate its expression, and this regulation is blocked by BETi (Figure 7F). YAP1 overexpression is sufficient to rescue the proliferation defect caused by BRD4 depletion, suggesting that YAP1 is a critical downstream target of BRD4. Furthermore, the BET inhibitor NHWD-870 acts synergistically with the MEK inhibitor trametinib to suppress melanoma growth, suggesting a promising combination-therapy strategy for melanoma treatment.

The oncogenic role of BRD4 was first discussed when BRD4-NUT fusion was shown to initiate the development of NUT-midline carcinoma 27. The BRD4-NUT fusion activates prosurvival genes such as MYC to facilitate the proliferation of NUT-midline carcinoma 28. Preclinical and clinical studies have demonstrated the efficacy of BET inhibition in several hematological malignancies 29, 30, in which inhibition of BRD4 leads to downregulation of MYC expression 31, a master regulator of cell survival and proliferation 32. However, the functional targets of BRD4 vary in other types of cancer. For example, the BET inhibitor JQ1 sensitized tumor cells to radiotherapy by regulating p21 in NSCLC 18. In prostate cancer, the BET inhibitor PFI-1 inhibits prostate cancer cell growth by suppressing the transactivation of full-length AR 19. In melanoma, a previous study failed to rescue the growth-inhibitory phenotype of BRD4 deletion with MYC overexpression 33, suggesting that MYC is not the main target of BRD4 in melanoma. Here, we demonstrated a strong correlation between BRD4 and YAP1 in melanoma patient samples. BRD4 bound to the *YAP1* promoter, and overexpression of YAP1 successfully rescued the proliferation suppression caused by BRD4 knockout. Together, these results suggest that YAP1 is a main downstream effector of BRD4 in melanoma.

BET inhibition has emerged as an appealing avenue for cancer treatment 34. Several clinical trials have shown promising results in some hematological malignancies, but the efficacy in solid tumors is limited due to low potency and intrinsic resistance 35. Efforts have been made to address this limitation by combining BETi with other therapies. One study set out to find an effective combination therapy with BET inhibition by screening approximately 1900 small molecular inhibitors. That study revealed that PI3K inhibitors are the most potent partners against neuroblastoma *in vitro* and *in vivo* 36. Another study also demonstrated that this combination can induce cell death and tumor regression in some breast cancer models, providing a promising strategy for fighting kinase inhibitor therapy resistance 37. Moreover, in an ovarian cancer model, combination treatment with PI3K inhibitors and BET inhibitors was shown to suppress the proliferation of cancer cells resistant to MEK inhibition 38. Other interesting possibilities for combination treatment are HDAC inhibitors, which can elevate the global level of histone acetylation, facilitating BET protein binding to chromatin. The synergistic effects have been demonstrated in a MYC-driven lymphoma model and a neuroblastoma model 39, 40. Here, we found that resistance to MEK inhibitors may be due to YAP1 upregulation, which can be overcome by BETi-mediated suppression of YAP1 expression. Furthermore, the combination of BETi and MEKi acts synergistically to suppress melanoma growth. Our results are consistent with those of previous studies on BETi and MEKi combinations in esophageal cancer and triple-negative breast cancer 41, 42. Together, these results suggest that the combination of BETi and MEKi is a promising therapeutic strategy against multiple solid tumors.

## Supplementary Material

Supplementary figures and table.

## Abbreviations

YAP1: Yes1-associated transcriptional regulator
BET inhibitor: the bromodomain and extra-terminal inhibitor
BRD2: Bromodomain Containing 2
BRD3: Bromodomain Containing 3
BRD4: Bromodomain Containing 4
BRDT: Bromodomain testis-specific protein
ChIP-Seq: Chromatin Immunoprecipitation Sequencing
