# Supplementary material for: Combined BET and MEK Inhibition synergistically suppresses melanoma by targeting YAP1
Source: Theranostics. 2024 Jan 1;14(2):593–607. doi: 10.7150/thno.85437 (PMC10758063; doi:10.7150/thno.85437)
Supplement: Supplementary file 1 — Supplementary figures and table. [file thnov14p0593s1.pdf]

Materials and methods

Compounds

NHWD-870 and BMS-986158 were synthesized by Ningbo Wenda Pharma (Ninghai, Zhejiang, China) and donated. JQ1 was purchased from MedChem Express (Cat # HY-13030). The other 101 small molecules were purchased from Selleck (Table S1).

Cell culture

A375 and SK-MEL-28 cells were obtained from the ATCC (Manassas, USA). Both were cultured in DMEM supplemented with 10% FBS, 100 U/mL penicillin and 100 mg/mL streptomycin. YUSOC, YUGASP, YUAME, and YUMAC are cell lines derived from tumors of patients treated at Yale University and were grown in Opti-MEM plus with 5% FBS.

Clinical samples

All clinical specimens in this study were collected with informed consent for research use and were approved by Central South University Institutional Review Boards in accordance with the Declaration of Helsinki. Melanoma tumor specimens were excised to alleviate tumor burden. The data referenced in this study are available under GSE190113 in the Gene Expression Omnibus (GEO). After institutional review board approval, we collected melanoma tissues from patients who met our inclusion criteria from January 2017 to December 2020 in the Tumor Hospital of Harbin Medical University. All the patients enrolled for the study were ≥18 years and had histologically

confirmed unresectable stage IIIC or IV melanoma with a mutation at the 600th position in BRAF. “Good outcome” and “Poor outcome” means the patients were still alive or dead when we collected the date in May, 2021.

Immunofluorescence study

Immunofluorescence staining of melanoma tumor sections was performed according to the manufacturer’s instructions. Quantitative comparison of YAP1 (ab56701, Abcam,1:400), and BRD4 (#13440, CST 1:200) expression was performed using freeware image analysis software (ImageJ, WS Rasband, National Health Institute, Bethesda, MA, USA) as previously reported [24]. The cell area was determined by manual delineation of raw fluorescence images. A minimum of 12 cells were analyzed from two independent experiments.

CRISPR/siRNAs knockout/knockdown and YAP1 overexpression

Knockout sgRNAs were designed according to online software CHOPCHOP (https://chopchop.rc.fas.harvard.edu/) and cloned into LentiCRISPRv2 vector. lentiviral plasmid, psPAX2, and pMD2.G were transfected into HEK293T cells in 6-well plates by Turbofect transfection Reagent (R0532, Thermo Fisher Scientific). The supernatants were collected and filtered (SLHV033RS, Millipore) 48 h after transfection. Lentiviruses were used for melanoma cell infection. Melanoma cells with stable knockdown or overexpression were selected with 1 μg/ml puromycin. YAP1-V5 in pLX304 (Addgene #25890) was used for YAP1 overexpression. Duplexes of siRNA

were synthesized by Genepharma (Shanghai, China). Transfection of siRNA was performed according to manufacturer's instructions. Non-targeting siRNA was used as a control.

The sequences of the sgRNAs were as follows:

BRD4: 1: 5'-AGACCAACCAACTGCAATACCT-3' and

2: 5'-GAGTCTGGGATGTTCGTCTCTC-3'; and

YAP1: 1: 5'-GTGCACGATCTGATGCCCGG-3' and

2: 5'-ACATCGATCAGACAACAACA-3'.

The sequences of the siRNAs were as follows:

YAP1: 1: sense 5'-CUGCCACCAAGCUAGAUAATT-3',

anti-sense 5'-UUAUCUAGCUUGGUGGCAGTT-3'; and

YAP1: 2: sense 5'-GCAUCUUCGACAGUCUUCUTT-3'

anti-sense 5'- AGAAGACUGUCGAAGAUGCTT-3'

RT–qPCR

Total RNA was isolated with TRIzol reagent (15596026, Invitrogen) according to the manufacturer's instructions. cDNA was synthesized with HiScript® II Q RT SuperMix for qPCR (+gDNA Wiper) following the manufacturer's instructions (R223-01, Vazyme). RT-qPCR was performed with 2X SYBR Green qPCR Master Mix (B21703, Bimake) using an ABI QuantStudio 3 PCR system. The expression of RNA is shown relative to the level of GAPDH mRNA. The primers used for RT–qPCR were as follows:

BRD2 F' GAGGTGTCCAATCCCAAAAAGC

BRD2 R'	ATGCGAACTGATGTTTCCACA
BRD3 F'	TCAAATTGAACCTGCCGGATT
BRD3 R'	TGCATACATTCGCTTGCACTC
BRD4 F'	CGCTATGTCACCTCCTGTTTGC
BRD4 R'	ACTCTGAGGACGAGAAGCCCTT
GAPDH F'	CTCTGCTCCTCCTGTTCGAC
GAPDH R'	GCCCAATACGACCAAATCC
YAP1 F'	TAGCCCTGCGTAGCCAGTTA
YAP1 R'	TCATGCTTAGTCCACTGTCTGT

Western blot assay

Whole-cell lysates were extracted with RIPA lysis buffer according to the manufacturer’s protocol. Proteins were separated on a 10% SDS–PAGE gel and identified by the following antibodies: an α-Tubulin (11H10) rabbit mAb (#2125, CST), an anti-YAP1 antibody (ab56701, Abcam), a c-Myc antibody (9E10, Novus Biologicals), a GAPDH (D4C6R) mouse mAb (#97166, CST), and a BRD4 (E2A7X) rabbit mAb (#13440, CST).

ChIP-seq

Chromatin immunoprecipitation (ChIP)-seq was performed by Acegen. Briefly, A375 cells were treated with 4 nM NHWD-870 for 3 days. Then, those cells were collected and cross-linked in DMEM with 1% formaldehyde. A Bioruptor was used to sonicate

chromatin, after which incubation was performed with a BRD4 antibody (#13440, CST) for immunoprecipitation. The DNA library was prepared using an Acegen DNA Library Prep Kit from Illumina, amplified by twelve-cycle PCR, cleaned up, analyzed with an Agilent 2100 Bioanalyzer and finally sequenced on the Illumina platform.

Colony formation assay and cell proliferation assay

For the colony formation assay, A375 cells or A375-derived cells were seeded into 6-well plates at 1000 cells per well. Seven days later, 10% formalin was used to fix the cells for 20 minutes at room temperature. Then, they were stained with 0.05% crystal violet in distilled water for 1 hour. Afterward, they were washed with water 3 times. Pictures were captured using a scanner. A CCK-8 assay was used to assess cell proliferation. A375 cells or SK-MEL-28 cells were seeded into 96-well plates at 800 cells per well and incubated for 1-5 days. At various time points, the medium was replaced with fresh medium containing 10% CCK-8 reagent (Bimake, Cat# B34302, USA). After incubation for 2.5 h, the absorbance at 450 nm was measured. For combinational treatments, the combination index (CI) was calculated using CompuSyn software (ComboSyn Inc., Biosoft; Cambridge, UK).

Ki67-positive cell analysis

A375 cells were seeded into 6-well plates at 5×10^5 cells per well. After 12 h, the cells were treated with NHWD-870 and/or trametinib. After incubation for 3 days, an anti-Ki67–FITC antibody (Thermo Fisher Scientific, #11-5698-80) was used to stain the

cells. Ki67-positive cells were detected by flow cytometry and analyzed using FlowJo software.

Animal studies

A total of $1x10^6$ A375- or A375-derived cells were subcutaneously implanted into the flanks of female nude mice. For drug treatment, when the tumors reached approximately 100 mm^3, 0.5 mg/kg NHWD-870 once daily (5 days on, 2 days off) and/or 0.5 mg/kg trametinib once daily or vehicle was administered to the mice orally [20, 25, 26]. The tumor sizes were measured every 3 or 4 days using a caliper. All mice were sacrificed by cervical dislocation after intraperitoneal injection with 0.1% pentobarbital sodium (30 mg/kg), and the tumors were dissected and fixed in formalin. All animals were housed in the specific pathogen-free animal facilities of Central South University with a 12:12 h light-dark cycle at a constant room temperature (22 ±1 °C) and fed a standard laboratory diet.

Figure S1

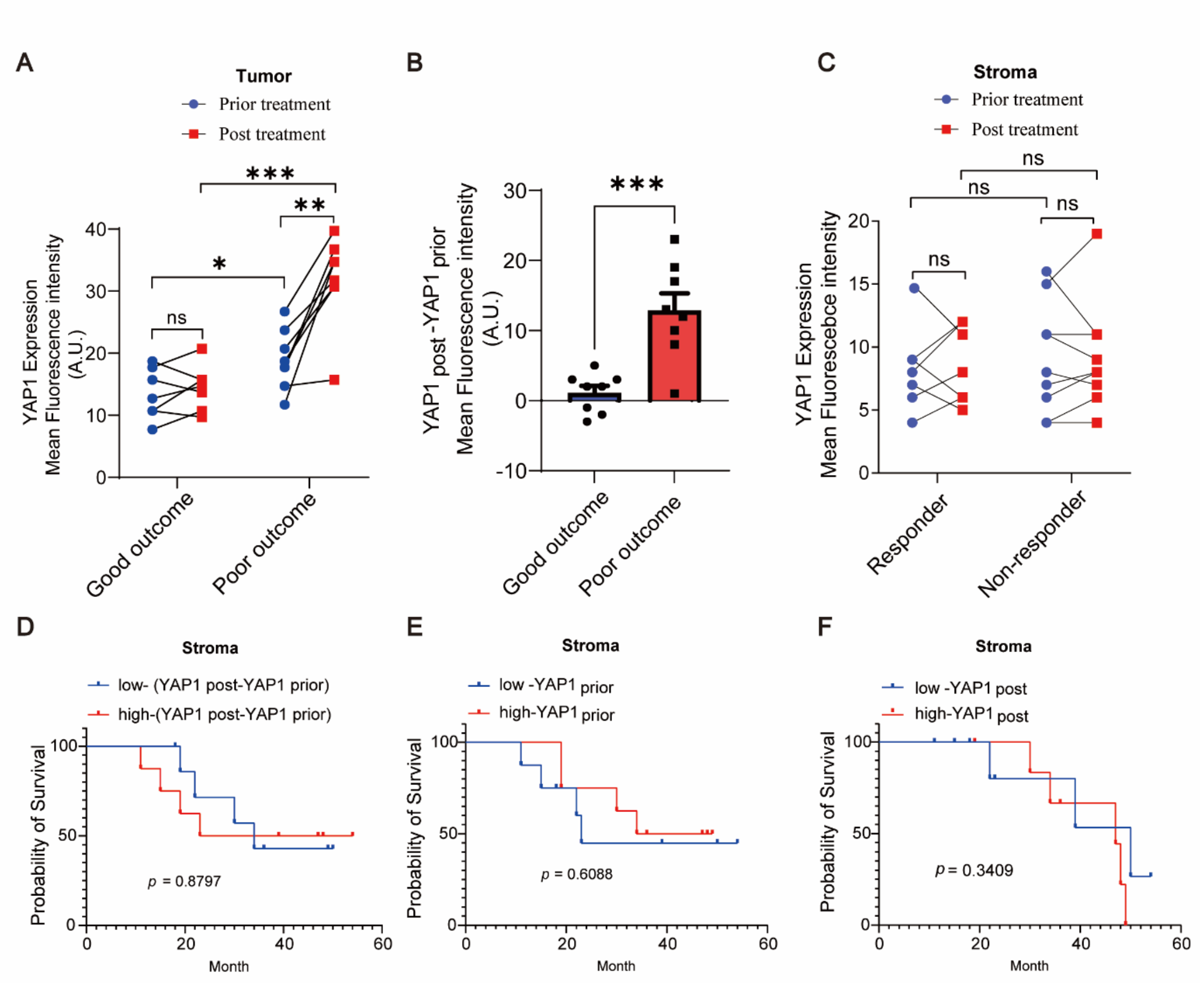

Figure S2

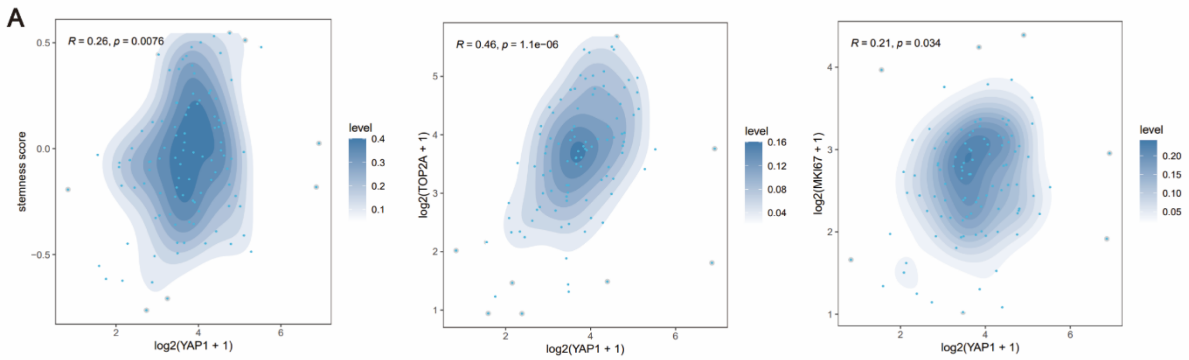

Figure S3

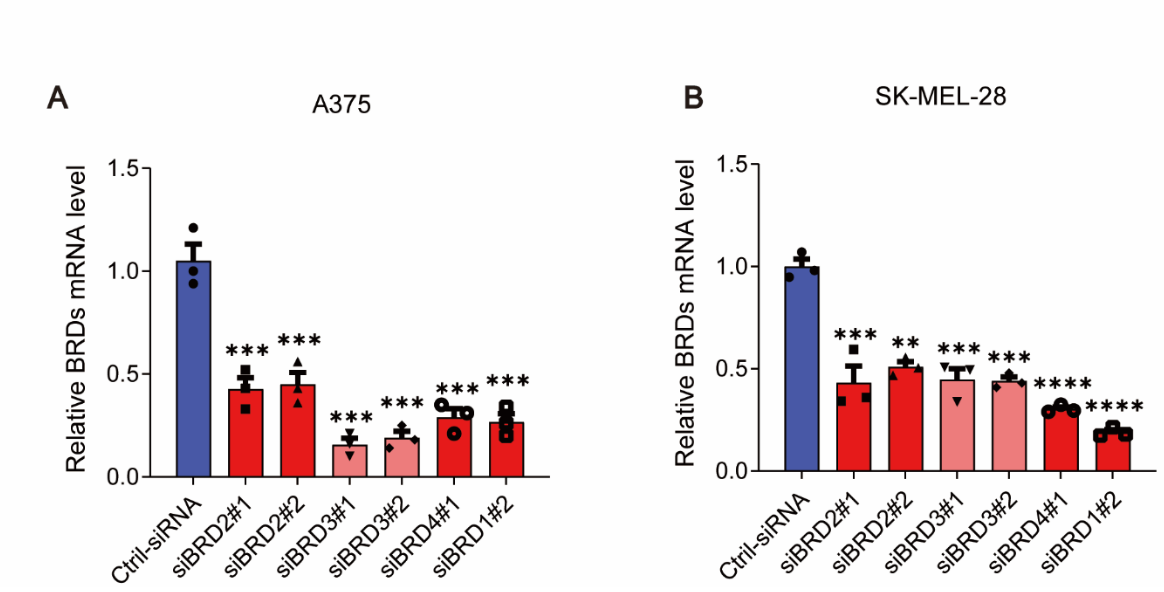

Figure S4

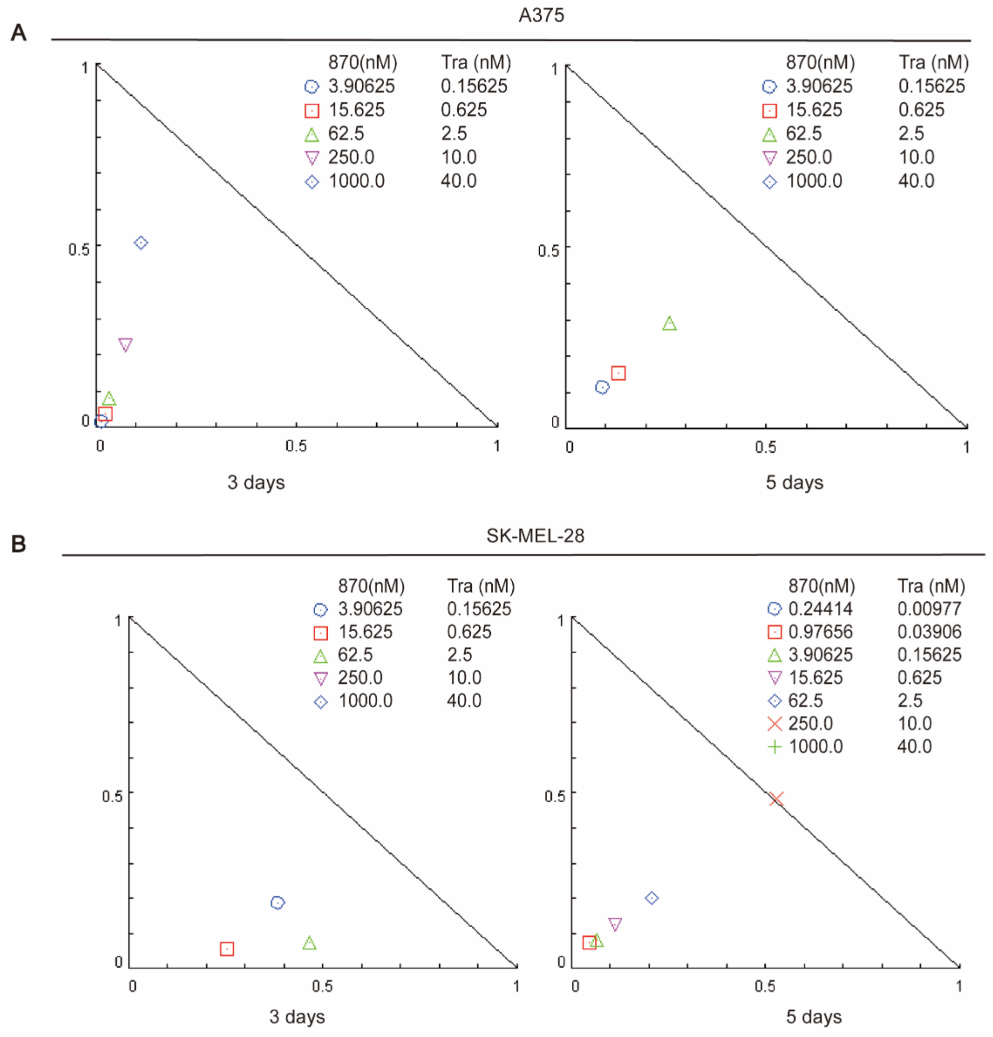

Figure S5

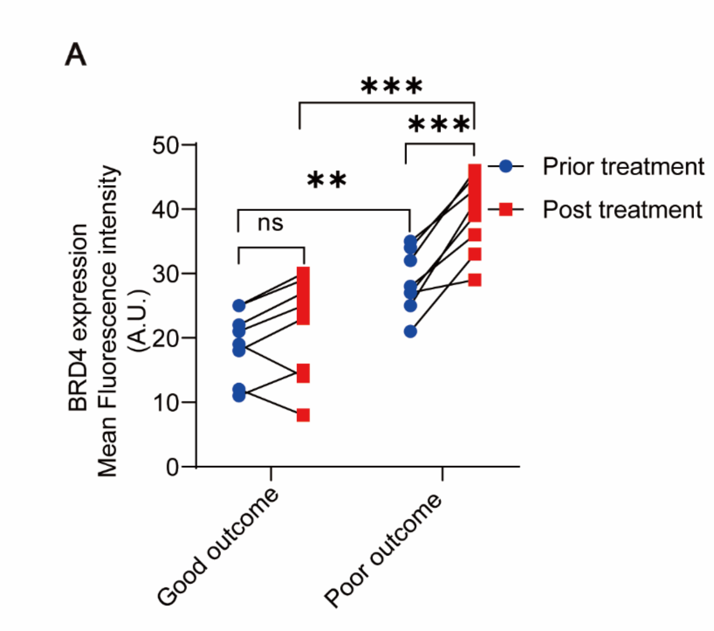

A
B
***
***
**
ns
50
40
30
20
10
0
BRD4 expression
Mean Fluorescence intensity
(A.U.)
Prior treatment
Post treatment
Good outcome
Poor outcome
**
20
15
10
5
0
-5
-10
BRD4 post-BRD4 prior
Mean Fluorescence intensity
(A.U.)
Good outcome
Poor outcome

Table S1

Table S1. Compounds information.

	cat	name	Indication	Target	M.w.	CAS Number	Formula	Form
1	S3612	Rosmarinic acid	Inflammation	IκB/IKK	360.31	20283-92-5	C18H16O8	free base
2	S3635	Medroxyprogesterone	Endocrinology	Estrogen/progestogen Receptor	344.49	520-85-4	C22H32O3	free base
3	S3657	Promestriene	Infection	Estrogen/progestogen Receptor	328.49	39219-28-8	C22H32O2	free base
4	S3671	Quinestrol	Endocrinology	Estrogen/progestogen Receptor	364.52	152-43-2	C25H32O2	free base
5	S3703	Phenethyl alcohol	Infection	Others	122.16	60-12-8	C8H10O	free base
6	S3714	Lifitegrast	Others	Integrin	615.48	1025967-78-5	C29H24Cl2N2O7S	free base
7	S3726	Selexipag	Cardiovascular Disease/Respiratory Disease	Immunology & Inflammation related	496.62	475086-01-2	C26H32N4O4S	free base
8	S3615	Dehydrocostus Lactone	Inflammation	IκB/IKK	230.30	477-43-0	C15H18O2	free base
9	S3785	Notoginsenoside R1	Others	Others	933.13	80418-24-2	C47H80O18	free base
10	S3659	Fludrocortisone acetate	Imflammation	Others	422.49	514-36-3	C23H31FO6	free base
11	S3672	Cefonicid sodium	Infection	Anti-infection	586.53	61270-78-8	C18H16N6Na2O8S3	disodium salt
12	S3704	4-Methylbenzylidene camphor	other	Others	254.37	36861-47-9	C18H22O	free base
13	S3716	Flibanserin	Endocrinology	5-HT Receptor	390.40	167933-07-5	C20H21F3N4O	free base
14	S3727	Vilanterol Trifenate	Respiratory Disease	Adrenergic Receptor	774.77	503070-58-4	C44H49Cl2NO7	trifenatate
15	S3738	Travoprost	Others	Others	500.55	157283-68-6	C26H35F3O6	free base
16	S3616	Asiaticoside	Inflammation	ROS	959.12	16830-15-2	C48H78O19	free base
17	S3637	Cefpirome sulfate	Infection	Anti-infection	612.66	98753-19-6	C22H24N6O9S3	sulfate
18	S3663	Afloqualone	Neurological Diseases	GABA Receptor	283.30	56287-74-2	C16H14FN3O	free base
19	S3705	Chlorobutanol	Infection	Anti-infection	177.46	57-15-8	C4H7Cl3O	free base
20	S3717	Testosterone Enanthate	Endocrinology	Androgen Receptor	400.59	315-37-7	C26H40O3	free base
21	S3728	Grazoprevir	Infection	HCV Protease	766.90	1350514-68-9	C38H50N6O9S	free base
22	S3739	Calcipotriene	Metabolic Disease	Others	412.60	112965-21-6	C27H40O3	free base
23	S3618	Acetylspiramycin (ASPM)	Infection	Anti-infection	885.09	24916-51-6	C45H76N2O15	free base
24	S3638	Cefamandole nafate	Infection	Anti-infection	512.49	42540-40-9	C19H17N6NaO6S2	sodium salt
25	S3664	Flupenthixol dihydrochloride	Neurological Disease	Dopamine Receptor,Adrenergic Receptor	507.44	51529-01-2	C23H27Cl2F3N2OS	dihydrochloride
26	S3817	Harmine hydrochloride	Others	DYRK	248.71	343-27-1	C13H13ClN2O	hydrochloride
27	S3706	Sarpogrelate hydrochloride	Neurological Diseases	5-HT Receptor	465.97	135159-51-2	C24H32ClNO6	hydrochloride
28	S3718	Leuprolide Acetate	Cancer	Estrogen/progestogen Receptor	1269.45	74381-53-6	C61H88N16O14	acetate

29	S3729	Iron sucrose	Others	Others	736.06	8047-67-4	C18H24Fe2O24	Iron saccharate
30	S3741	Benznidazole	Infection	Anti-infection	260.25	22994-85-0	C12H12N4O3	free base
31	S3621	Pazufloxacin mesylate	Infection	Anti-infection	414.41	163680-77-1	C17H19FN2O7S	methanesulfonate
32	S3639	Tacrine hydrochloride hydrate	Neuronal Signaling	AChR	198.26	206658-92-6	C13H14N2R2	hydrochloride hydrate
33	S3666	Ilaprazole	Inflammation	Proton Pump	366.44	172152-36-2	C19H18N4O2S	free base
34	S3694	Glucosamine hydrochloride	Others	Others	215.63	66-84-2	C6H14ClNO5	hydrochloride
35	S3707	Ethopabate	Infection	Anti-infection	237.25	59-06-3	C12H15NO4	free base
36	S3719	Topiroxostat	Metabolic Disease	Others	248.24	577778-58-6	C13H8N6	free base
37	S3730	Metaxalone	Neurological Diseases	Others	221.25	1665-48-1	C12H15NO3	free base
38	S3742	Cholic acid	Metabolic Disease	Others	408.57	81-25-4	C24H40O5	free base
39	S3622	Diammonium Glycyrrhizinate	Inflammation	Immunology & Inflammation related	856.99	79165-06-3	C42H68N2O16	ammonium salt
40	S3640	Methoxyphenamine Hydrochloride	Respiratory Disease	Adrenergic Receptor	215.72	5588-10-3	C11H18ClNO	hydrochloride
41	S3835	Loganin	Others	BACE,AChR	390.38	18524-94-2	C17H26O10	free base
42	S3668	Thymopentin	other	Immunology & Inflammation related	679.77	69558-55-0	C30H49N9O9	free base
43	S3697	Mafenide hydrochloride	Infection	Carbonic Anhydrase	222.69	138-37-4	C7H11ClN2O2S	hydrochloride
44	S3708	Sulfachloropyridazine	Infection	Anti-infection	284.72	80-32-0	C10H9ClN4O2S	free base
45	S3722	Isavuconazole	Infection	Anti-infection	437.47	241479-67-4	C22H17F2N5OS	free base
46	S3731	Tipiracil hydrochloride	Cancer	Phosphorylase	279.12	183204-72-0	C9H12Cl2N4O2	hydrochloride
47	S3623	Ceftibuten dihydrate	Microbiology	Anti-infection	446.46	118081-34-8	C15H18N4O8S2	0
48	S3641	Osalmid	Inflammation	Others	229.23	526-18-1	C13H11NO3	free base
49	S3669	Carmustine	Cancer	DNA/RNA Synthesis	214.05	154-93-8	C5H9Cl2N3O2	free base
50	S3698	Nortriptyline hydrochloride	Neurological Diseases	Others	299.84	894-71-3	C19H22ClN	hydrochloride
51	S3711	Carbasalate Calcium	Inflammmation	Others	458.43	5749-67-7	C19H18CaN2O9	calcium salt
52	S3723	Ramosetron Hydrochloride	Gastroenterology	5-HT Receptor	315.80	132907-72-3	C17H18ClN3O	hydrochloride
53	S3732	Avibactam sodium	Infection	Anti-infection	287.23	1192491-61-4	C7H10N3NaO6S	sodium salt
54	S3745	Balsalazide disodium	Inflammation	Immunology & Inflammation related	437.31	150399-21-6	C17H17N3Na2O8	disodium salt dihydrate
55	S3643	Amitraz	Infection	Adrenergic Receptor	293.41	33089-61-1	C19H23N3	free base
56	S3670	Cefsulodin sodium	Infection	phosphatase	554.53	52152-93-9	C22H19N4NaO8S2	sodium salt

57	S3701	Benactyzine hydrochloride	Neurological Diseases	AChR	363.88	57-37-4	C20H26ClNO3	hydrochloride
58	S3713	Moxidectin	Microbiology	Anti-infection	639.82	113507-06-5	C37H53NO8	free base
59	S3724	Velpatasvir	Infection	HCV Protease	883.00	1377049-84-7	C49H54N8O8	free base
60	S3733	Boceprevir	Infection	HCV Protease	519.68	394730-60-0	C27H45N5O5	free base
61	S3746	Lumefantrine	Infection	Anti-infection	528.94	82186-77-4	C30H32Cl3NO	free base
62	S3779	cis-Anethole	Others	Others	148.20	104-46-1	C10H12O	free base
63	S3747	Levothyroxine sodium	Endocrinology	Others	798.85	55-03-8	C15H10I4NNaO4	sodium salt
64	S3763	Cinnamaldehyde	Others	TRPV	132.16	14371-10-9	C9H8O	free base
65	S3783	Echinacoside	Others	Others	786.73	82854-37-3	C35H46O20	free base
66	S3809	Imperatorin	Others	Calcium Channel	270.28	482-44-0	C16H14O4	free base
67	S3847	Panaxatriol	other	Others	476.73	32791-84-7	C30H52O4	free base
68	S3750	Sodium benzoate	Neurological Diseases	Others	144.10	532-32-1	C7H5NaO2	sodium salt
69	S3766	Tanshinone IIA sulfonate (sodium)	other	CaMK	396.39	69659-80-9	C19H17NaO6S	sodium salt
70	S3810	Scutellarin	Others	Others	462.36	27740-01-8	C21H18O12	free base
71	S3849	D-Galactose	other	Others	180.16	59-23-4	C6H12O6	free base
72	S3751	Quinidine sulfate	Cardiovascular Disease	Sodium Channel	648.83	6591-63-5	C40H54N4O10S	sulfate dihydrate
73	S3769	Palmatine	Infection	AChR	352.40	3486-67-7	C21H22NO4+	free base
74	S3788	Carvacrol	Others	Others	150.22	499-75-2	C10H14O	free base
75	S3811	Ginsenoside Re	Others	Others	947.15	52286-59-6	C48H82O18	free base
76	S3850	Glucosamine sulfate	Others	Others	277.25	29031-19-4	C6H15NO9S	sulfate
77	S3754	4-Hydroxybenzoic acid	Infection	Others	138.12	99-96-7	C7H6O3	free base
78	S3772	5-Hydroxymethylfurfural	Others	Others	126.11	67-47-0	C6H6O3	free base
79	S3791	Succinic acid	other	Others	118.09	110-15-6	C4H6O4	free base
80	S3851	Camphor	Inflammation	Others	152.23	76-22-2	C10H16O	free base
81	S3755	Betaine	Others	Others	117.15	107-43-7	C5H11NO2	inner salt
82	S3773	Tyrosol	other	Others	138.16	501-94-0	C8H10O2	free base
83	S3794	Palmitic acid	other	Others	256.42	57-10-3	C16H32O2	free base
84	S3824	Quercitrin	Others	Immunology & Inflammation related	448.38	522-12-3	C21H20O11	free base
85	S3854	Tetrahydropalmatine hydrochloride	Neurological Disease	Others	391.89	6024-85-7	C21H26ClNO4	hydrochloride
86	S3756	Methyl salicylate	Transmembrane Transporters	TRPV	152.15	119-36-8	C8H8O3	free base
87	S3775	Ligustrazine hydrochloride	other	Immunology & Inflammation related	172.66	76494-51-4	C8H13ClN2	hydrochloride
88	S3802	Trigonelli	Others	Others	173.60	6138-41-	C7H8ClN	chlorid

57	S3701	Benactyzine hydrochloride	Neurological Diseases	AChR	363.88	57-37-4	C20H26ClNO3	hydrochloride
58	S3713	Moxidectin	Microbiology	Anti-infection	639.82	113507-06-5	C37H53NO8	free base
59	S3724	Velpatasvir	Infection	HCV Protease	883.00	1377049-84-7	C49H54N8O8	free base
60	S3733	Boceprevir	Infection	HCV Protease	519.68	394730-60-0	C27H45N5O5	free base
61	S3746	Lumefantrine	Infection	Anti-infection	528.94	82186-77-4	C30H32Cl3NO	free base
62	S3779	cis-Anethole	Others	Others	148.20	104-46-1	C10H12O	free base
63	S3747	Levothyroxine sodium	Endocrinology	Others	798.85	55-03-8	C15H10I4NNaO4	sodium salt
64	S3763	Cinnamaldehyde	Others	TRPV	132.16	14371-10-9	C9H8O	free base
65	S3783	Echinacoside	Others	Others	786.73	82854-37-3	C35H46O20	free base
66	S3809	Imperatorin	Others	Calcium Channel	270.28	482-44-0	C16H14O4	free base
67	S3847	Panaxatriol	other	Others	476.73	32791-84-7	C30H52O4	free base
68	S3750	Sodium benzoate	Neurological Diseases	Others	144.10	532-32-1	C7H5NaO2	sodium salt
69	S3766	Tanshinone IIA sulfonate (sodium)	other	CaMK	396.39	69659-80-9	C19H17NaO6S	sodium salt
70	S3810	Scutellarin	Others	Others	462.36	27740-01-8	C21H18O12	free base
71	S3849	D-Galactose	other	Others	180.16	59-23-4	C6H12O6	free base
72	S3751	Quinidine sulfate	Cardiovascular Disease	Sodium Channel	648.83	6591-63-5	C40H54N4O10S	sulfate dihydrate
73	S3769	Palmatine	Infection	AChR	352.40	3486-67-7	C21H22NO4+	free base
74	S3788	Carvacrol	Others	Others	150.22	499-75-2	C10H14O	free base
75	S3811	Ginsenoside Re	Others	Others	947.15	52286-59-6	C48H82O18	free base
76	S3850	Glucosamine sulfate	Others	Others	277.25	29031-19-4	C6H15NO9S	sulfate
77	S3754	4-Hydroxybenzoic acid	Infection	Others	138.12	99-96-7	C7H6O3	free base
78	S3772	5-Hydroxymethylfurfural	Others	Others	126.11	67-47-0	C6H6O3	free base
79	S3791	Succinic acid	other	Others	118.09	110-15-6	C4H6O4	free base
80	S3851	Camphor	Inflammation	Others	152.23	76-22-2	C10H16O	free base
81	S3755	Betaine	Others	Others	117.15	107-43-7	C5H11NO2	inner salt
82	S3773	Tyrosol	other	Others	138.16	501-94-0	C8H10O2	free base
83	S3794	Palmitic acid	other	Others	256.42	57-10-3	C16H32O2	free base
84	S3824	Quercitrin	Others	Immunology & Inflammation related	448.38	522-12-3	C21H20O11	free base
85	S3854	Tetrahydropalmatine hydrochloride	Neurological Disease	Others	391.89	6024-85-7	C21H26ClNO4	hydrochloride
86	S3756	Methyl salicylate	Transmembrane Transporters	TRPV	152.15	119-36-8	C8H8O3	free base
87	S3775	Ligustrazine hydrochloride	other	Immunology & Inflammation related	172.66	76494-51-4	C8H13ClN2	hydrochloride

88	S3802	Trigonelline Hydrochloride	Others	Others	173.60	6138-41-6	C7H8ClNO2	chloride
89	S3856	Allantoin	other	Others	158.12	97-59-6	C4H6N4O3	free base
90	S3758	Sinomenine hydrochloride	Inflammation	Immunology & Inflammation related,Autophagy	365.85	6080-33-7	C19H24ClNO4	hydrochloride
91	S3805	Stevioside	Others	Others	804.87	57817-89-7	C38H60O18	free base
92	S3842	Isoquercitrin	Others	Wnt/beta-catenin	464.38	482-35-9	C21H20O12	free base
93	S3858	Lawsone	Infection	Others	174.15	83-72-7	C10H6O3	free base
94	S3761	Eucalyptol	Inflammation	Immunology & Inflammation related	154.25	470-82-6	C10H18O	free base
95	S3781	Ginkgolide C	Others	Others	440.40	15291-76-6	C20H24O11	free base
96	S3807	Dehydroandrographolide	Inflammation	Chloride Channel	332.43	134418-28-3	C20H28O4	free base
97	S3843	Madecassoside	Neurological Disease	Others	975.12	34540-22-2	C48H78O20	free base
98	S3866	Galanthamine	Neurological Disease	AChR	287.35	357-70-0	C17H21NO3	free base
99	S7110	JQ1	Others	BRDs	456.99	1268524-70-4	C23H25ClN4O2S	free base
100	S7360	Birabresib (OTX015)	Others	BRDs	491.99	202590-98-5	C25H22ClN5O2S	free base
101	S9691	BMS-986158	Solid Tumor Childhood|Lymphoma|Brain Tumor Pediatric	BRDs	495.62	1800340-40-2	C30H33N5O2	free base
